# The Glycobiology of Pulmonary Arterial Hypertension

**DOI:** 10.3390/metabo12040316

**Published:** 2022-04-01

**Authors:** Shia Vang, Phillip Cochran, Julio Sebastian Domingo, Stefanie Krick, Jarrod Wesley Barnes

**Affiliations:** Division of Pulmonary, Allergy and Critical Care Medicine, Department of Medicine, University of Alabama at Birmingham, Birmingham, AL 35294, USA; svang35@uab.edu (S.V.); prcochran@yahoo.com (P.C.); julio10@uab.edu (J.S.D.); skrick@uabmc.edu (S.K.)

**Keywords:** pulmonary hypertension, glycobiology, metabolism

## Abstract

Pulmonary arterial hypertension (PAH) is a progressive pulmonary vascular disease of complex etiology. Cases of PAH that do not receive therapy after diagnosis have a low survival rate. Multiple reports have shown that idiopathic PAH, or IPAH, is associated with metabolic dysregulation including altered bioavailability of nitric oxide (NO) and dysregulated glucose metabolism. Multiple processes such as increased proliferation of pulmonary vascular cells, angiogenesis, apoptotic resistance, and vasoconstriction may be regulated by the metabolic changes demonstrated in PAH. Recent reports have underscored similarities between metabolic abnormalities in cancer and IPAH. In particular, increased glucose uptake and altered glucose utilization have been documented and have been linked to the aforementioned processes. We were the first to report a link between altered glucose metabolism and changes in glycosylation. Subsequent reports have highlighted similar findings, including a potential role for altered metabolism and aberrant glycosylation in IPAH pathogenesis. This review will detail research findings that demonstrate metabolic dysregulation in PAH with an emphasis on glycobiology. Furthermore, this report will illustrate the similarities in the pathobiology of PAH and cancer and highlight the novel findings that researchers have explored in the field.

## 1. Introduction

Pulmonary vascular diseases (PVDs) are a group of pulmonary ailments resulting from precapillary or postcapillary hypertension and include pulmonary embolism, chronic thromboembolic disease, pulmonary veno-occlusive disease, arteriovenous malformations, pulmonary edema, and pulmonary hypertension (PH) [1,2]. Several clinical studies have estimated that PVDs occur in 20–25 million persons worldwide; however, the occurrence as well as the pathogenesis still remain unknown. Currently, PH is the most complex and widely studied PVD and is therefore the focus of this review. 

In 2009, PH was defined by a mean pulmonary artery pressure (mPAP) of ≥25 mm Hg at rest that is measured during right heart catheterization (RHC) [3,4]. It was recently recommended to lower the mPAP threshold to ≥20 mm Hg in 2019 based on scientific studies [5,6]. PH is often associated with other PVDs or diseases such as congenital heart disease, coronary artery disease, liver cirrhosis, and COPD, as well as personal and environmental factors such as tobacco use, drug use, and toxin stimuli. Therefore, PH has been classified by the World Health Organization (WHO) as a five-group system according to etiology: Group 1, PAH; Group 2, PH due to left heart disease; Group 3, PH due to chronic lung disease or hypoxia; Group 4, Chronic Thromboembolic PH (CTEPH); and Group 5, PH due to unclear multifactorial mechanisms [5].

Pulmonary Arterial Hypertension (PAH), the Group 1 subcategory of PH, is characterized hemodynamically by the presence of pre-capillary PH with an end-expiratory pulmonary artery wedge pressure (PAWP) ≤ 15 mm Hg and a pulmonary vascular resistance >3 Wood units [7], which frequently leads to right ventricular (RV) hypertrophy/failure and early mortality. RHC remains essential for a diagnosis of IPAH [8]. From the time of diagnosis, PAH has an average survival of 2–3 years without treatment and affects mostly women; however, men diagnosed with PAH have lower survival rates [9,10]. The pathobiology of PAH is complex; features include vasoconstriction, inflammation, genetics/epigenetics, and/or environmental cues [11,12,13,14,15,16] as well as increased cell proliferation, vascular remodeling, and angiogenesis [17,18,19,20,21,22]. Furthermore, metabolic dysfunction that includes deficits in nitric oxide (NO) production [23,24,25,26,27], insulin resistance [28,29,30,31], leptin dysregulation [32,33,34], dyslipidemia, and increased lipid oxidation [35,36,37,38,39,40,41,42] have been established in IPAH and may affect disease progression. Currently, the primary therapies for PAH are those that target the mediators of vasoconstriction (e.g., NO, prostacyclin, or endothelin) [23,43,44,45], while other pathomechanisms including cell proliferation, increased angiogenesis, inflammation, and plexiform lesions (a hallmark of PAH) have no known treatment options. This review will discuss metabolic dysregulation with an emphasis on novel findings in glycobiology observed in Group 1 PAH, where the most of the investigations have been documented. 

### 1.1. Metabolism Studies in PAH

Over the years, evidence for the role of metabolites in PAH has been documented and determined to affect the many processes mentioned above. Other reports have shown that metabolites including glucose [30,46,47,48,49], ammonia [50], arginine [51,52], glutamine [53,54,55], and fatty acids are dysregulated [35,37,41,56]. Interestingly, altered glucose influx into cells can lead to glucose intolerance in PAH, and studies have shown that glycated hemoglobin levels correlate with idiopathic PAH (IPAH) diagnosis [30,57]. This suggests that chronic hyperglycemia may perpetuate glucose influx and insulin resistance in IPAH patients.

Several reports have also emphasized the similarities between cancer and PAH [58,59,60,61]. For example, cancer cells, such as PAH, display increased cellular glucose uptake and altered glucose metabolism [62,63,64,65]. Highly proliferative cancer cells require immense energy and utilize excess metabolites, a characteristic that has become a primary research focus in PAH. Highly proliferative cells have been postulated to alter their glycolytic metabolism rates and to shift from glucose oxidation and CO_2_ production to aerobic glycolysis despite normal oxygen levels (also known as the Warburg effect) [66,67]. Rapidly growing cells benefit from aerobic glycolysis because it improves their survival rate when total cell numbers increase, and the oxygen supply is drastically reduced. Highly proliferative cells, which switch to aerobic glycolysis, yield less ATP than those that utilize mitochondrial respiration (oxidative phosphorylation). In turn, these cells take up glucose more rapidly when levels are abundantly available.

Similar findings of vascular cell proliferation and changes in glucose metabolism have been determined in IPAH. In particular, (18F)-Fluoro-deoxyglucose-PET (FDG-PET) scans have shown that the lungs of patients with IPAH have increased uptake of glucose and have a higher glycolytic rate than healthy individuals [48,68]. In addition, IPAH endothelial cells display three-fold more glycolysis than normal cells, which contributes to an increased rate of growth and proliferation [68]. Other studies have shown that pulmonary vascular cells from several PH animal models and IPAH human tissue have hyperpolarized mitochondria and suppressed glucose oxidation and respiration. Glycolysis in PAH is enhanced, and mitochondrial dysfunction with reduced oxidative phosphorylation has also been observed in PAH (and cancer) [69,70,71,72,73,74]. Multiple reports have shown that a key enzyme, pyruvate dehydrogenase (PDH), which regulates pyruvate influx (a product of glycolysis) into the mitochondria, is inhibited and results in the suppression of glucose oxidation [72,75]. The role of PDH in PAH (and cancer) has been demonstrated through pharmacological inhibition with dichloroacetate (DCA), a known activator of PDH, and knockout of fatty acid metabolic enzymes [41,76,77,78,79]. Both of these interventions alter the ‘glycolytic shift’, augment glucose oxidation, and reverse PAH phenotypes in several models of PAH. In the PAH pulmonary vasculature, cells have been postulated to adapt to these metabolic alterations (nutrient stress conditions) by reprograming their metabolism and protein homeostasis, resulting in increased survival and proliferation.

### 1.2. Carbohydrate Metabolism and Glycosylation

Most of the proteins produced by the human body contain linked sugar chains or glycans, and all multicellular organisms utilize these molecules as biosignals in normal physiology [80,81,82]. Glycans are formed as secondary gene products by the concerted action of glycosyltransferases, glycosidases, and high energy sugar nucleotides (e.g., UDP-GlcNAc, UDP-GalNAc, and CMP-sialic acid). Therefore, the biosynthesis of glycans is not controlled by an interventional template, and their structures are much less rigidly defined than those of proteins and nucleic acids. In addition, carbohydrates and glycans are dynamic and altered by the cellular microenvironment. This is exemplified by malignant cells in which metabolic dysregulation has been shown to alter glycan structures [83,84,85]. An investigation of glycosylation changes driven by disease has already produced some novel insights. In particular, changes in the molar proteoglycan (PG) ratios have been demonstrated in patients with aging skin and with arthritis [86,87]. In addition, altered glycan patterns of glycolipids have been linked to aging [88,89,90], and glycan changes on glycoproteins have also been shown in patients with Alzheimer’s disease [91,92,93,94,95,96,97] as well as cardiovascular disease [98,99,100], COPD [101,102,103,104,105,106], and other pulmonary diseases [107,108,109,110,111,112,113,114,115]. Studying glycans, carbohydrate precursors/pathways, and/or the glycan machinery (i.e., glycosyltransferases and glycosylhydrolases) may hold essential information that is critical for uncovering mechanism(s) in these metabolic diseases, including PAH, where these post-translational modifications have not been widely studied. 

### 1.3. Hexosamine Biosynthetic Pathway

The hexosamine biosynthetic pathway (HBP) has been documented as a cellular sensor for nutrient uptake [116,117,118,119]. Indeed, cells during proliferation use excess metabolites, namely glucose, glutamine, acetyl-CoA, and uridine triphosphate (UTP) to fuel their demanding energy requirements, all of which are also funneled into the HBP. This pathway is critical for the synthesis of the highly ‘energy’ charged sugar nucleotide, UDP-GlcNAc, which is one of the most fundamentally important building blocks for all glycosylation events. UDP-GlcNAc is also a precursor to other sugar nucleotides such as UDP-GalNAc and CMP-sialic acid that are essential for generating oligosaccharides, which make up all glycoconjugates (e.g., glycoproteins and proteoglycans) and glycosaminoglycans (GAGs). 

### 1.4. Intracellular Glycosylation (O-GlcNAc) in PAH

UDP-GlcNAc biosynthesis has been most studied for its role as the substrate/precursor for the O-linked N-Acetylglucosamine (O-GlcNAc) post-translational protein modification. The ‘GlcNAc’ moiety from UDP-GlcNAc is transferred and covalently attached to proteins on serine/threonine residues [120,121] through catalytic activity of the O-GlcNAc transferase (OGT). Conversely, the removal of ‘GlcNAc’ is performed by O-GlcNAc hydrolase (OGA) [122,123,124]. The O-GlcNAc modification is found throughout the cell including the cytoplasm, nucleus, and mitochondria [125,126,127]. O-GlcNAc has been demonstrated to have an antagonistic role on phosphorylation [128,129,130]. Since its discovery, more than 1900 articles have characterized protein O-GlcNAcylation with over 5000 human proteins identified to bear O-GlcNAc [131].

The addition/removal of O-GlcNAc is a dynamic process under the influence of the cellular environment (i.e., stress, hormones, and nutrient flux) [132,133,134,135,136,137,138]. For this reason, the O-GlcNAc modification has been well documented as a nutrient sensor and flux mediator [123,125,133,137,139]. Alterations in glucose uptake, similar to those found in IPAH, can regulate protein O-GlcNAc levels and are likely to influence protein activity/function. On the other hand, an imbalance in the OGT, OGA, and O-GlcNAc has been documented to impact glucose utilization in cancer metabolism [140,141,142,143,144,145]. The crux of O-GlcNAc modification is regulated by the concentration of UDP-GlcNAc sugar pools, which is governed by flux into the HBP, suggesting that the intrinsic mechanisms and regulators of the pathway may be controlled by O-GlcNAc. 

The O-GlcNAc modification of proteins in vascular disease has been described [100,135,146,147,148,149]. However, the exact role of the O-GlcNAc modification in vascular disease is still poorly understood. In particular, the role of this modification in PAH is just beginning to be recognized. We recently showed that alterations in glucose uptake and flux into the HBP led to augmented OGT expression/activity, increased proliferation in IPAH pulmonary artery smooth muscle cells (PASMCs), and faster time to clinical worsening in PAH (as defined by hospitalization, lung transplantation, or death; n = 86 PAH patients) [139]. In addition, we later demonstrated that reductions in O-GlcNAc levels contributed to impaired IPAH vascular sprouting and de novo vascularization [150] as well as alterations in eNOS activity and NO production [151]. Other studies followed our initial reports showing a connection between increased O-GlcNAc, RV glucose uptake, RV metabolic derangements, and RV function in PAH [152,153]. These findings indicate a direct role for the O-GlcNAc modification in PAH disease pathology.

### 1.5. The Role of Extracellular Matrix (ECM) Glycosaminoglycans (GAGs) in PAH

ECM is a dynamic scaffold with a fundamental role in regulating tissue and cellular function. The ECM is made up of proteins and PGs that greatly influence ECM development, stem cell differentiation, cellular morphology, and cell signaling [154]. In disease pathology, dysregulated ECM remodeling has been shown to contribute to dysfunctional cellular processes such as cell signaling, migration, proliferation, and adhesion. In particular, PGs are known to facilitate the aforementioned processes. In addition, PGs are proteins glycosylated with anionic GAGs [155,156,157,158,159,160,161]. GAG chains are typically long and unbranched with alternating disaccharide units of either D-glucuronic or L-iduronic acid and either GlcNAc or GalNAc. Often, one or both sugars contain sulfate moieties with the exception of hyaluronic acid (HA) that does not bear sulfation and is not covalently linked to a core protein. PGs are categorized by their GAG chain and fall into groups containing either heparan sulfate (HS), chondroitin sulfate (CS), dermatan sulfate (DS), or keratan sulfate (KS) [162]. Based on the vascular abnormalities (e.g., remodeling, intimal thickening, and plexiform lesion formation) observed in PAH, it is clear that altered PG biosynthesis/turnover may contribute to the pathogenesis of the disease.

In PAH, remodeling of the pulmonary vasculature and neovascularization are known consequences of disruptive changes to the ECM of vascular cells/tissue. Interestingly enough, hypoxia has been shown to potentiate GAG synthesis in human primary lung fibroblasts from normal lungs [163,164] and may offer similar regulation of GAG synthesis/deposition in PH. In PH animal models, ECM remodeling has been suggested to occur during early hypoxia exposure, which precedes increases in RV systolic pressure (RVSP) and RV hypertrophy [165,166]. Furthermore, hypoxia, vascular dysfunction, and dysregulated glucose metabolism, all of which are contributors to IPAH pathogenesis, have been suggested to modulate PG synthesis/turnover [167,168,169,170,171,172,173,174]. Several groups have identified specific PGs and their potential role in IPAH including HA, perlecan, versican, aggrecan, and syndecan. 

### 1.6. Hyaluronan (HA) in PAH

HA is a large GAG composed of an alternate repeat of two sugars, GlcNAc and glucuronic acid (GlcUA), and is found in the pericellular matrix and ECM [175]. HA also exists in the synovial fluid, dermis, and vitreous body [176]. HA is formed at the cell membrane by any of three HA synthase enzymes (HAS1, HAS2, or HAS3) by alternately adding GlcUA and GlcNAc to the reducing end of the nascent polysaccharide, which is extruded through the cell membrane into the extracellular space [177,178]. Conversely, the turnover/degradation of HA differs from tissue to tissue and is performed by the hyaluronidases (Hyal1 and Hyal2). Recent reports have shown that HA synthesis is controlled through cytosolic UDP-GlcNAc levels generated from the HBP [179,180]. Through the regulatory production of UDP-GlcNAc sugar pools, the HBP controls not only the intracellular energy state of the cell but also the extracellular integrity surrounding the cell through HA production. 

Multiple studies over the past few decades have contributed to our understanding of HA and its role in pulmonary health and disease [181,182,183,184]. Once thought to be only a scaffolding molecule of the ECM, research has shown that HA is an important active regulator of inflammation, airway hyperresponsiveness [185,186,187], lung injury [188,189,190], and fibrosis in the lung [112,113,188,191]. The presence of HA in IPAH has also been described. Elevated levels of HA in IPAH were first demonstrated by Aytekin et al. in 2008 [192]. They showed increased plasma HA levels in IPAH patients as well as abnormal levels in the plexogenic lesions and PASMCs. In addition, Lauer, Aytekin, et al. showed increased binding of inflammatory cells to a pathological form of HA (covalently modified with heavy chains from inter-alpha-inhibitor) in IPAH, suggesting that there may be a role for HA in regarding the processes of remodeling and inflammation in the disease [193]. Others showed similar findings, where HA content was elevated in IPAH compared to control donor lungs [194], as well as a potential role for endothelin-1 in HA synthesis and THP-1 monocyte adhesion [195].

Even though the role of HA has been recognized in PAH, its regulation and precise functional role in the disease still remains unknown. Cell culture models may hold the key to understanding the regulatory mechanisms associated with HA synthesis. In culture models, HA production can be induced by inhibitors of protein synthesis as well as the viral mimetic polyinosinic acid:polycytidylic (poly I:C) [196]. Similarly, the addition of high glucose to cell cultures can stimulate HA synthesis in cultured cell models [197]. A possible mechanism for the increase in HA levels in PASMCs may be due to the vast uptake of glucose [49,139], similar to the uptake described in PAECs [68]. HA, along with other GAGs, in PAH may be functionally involved in the remodeling process as well as the metabolic abnormalities that contribute to the overall disease pathology including angiogenesis, proliferation, and inflammation. 

### 1.7. Perlecan in PAH

Heparan sulfate proteoglycans (HSPGs) contain a core protein linked to HS GAG chains [154]. Examples of HSPGs are perlecan, agrin, type XVIII collagen α1, and glypican. HSPGs are located in the ECM and provide structural support as well as promote cell-to-cell and cell-to-matrix interactions, and cell motility and migration [198]. In addition, the HSPGs interact with cytokines, chemokines, morphogens, and growth factors while facilitating spatial/temporal cell signaling and morphogen gradient formation between cells and in tissues [155,157,199,200]. Perlecan, a major HSPG in the vasculature, is important in vasculogenesis and maintaining vascular tone [201,202]. Interestingly, both perlecan and agrin were found to be higher in PAH patients [203]. Most of the research that has been carried out has been conducted on perlecan, which has been observed in high abundance in junctions between PAH PAECs and PASMCs [204,205,206]. In addition, the role of perlecan in PAH may be linked to its involvement in EC barrier function, cell-to-cell interactions between PAECs and PASMCs, and inhibition of SMC proliferation [204], all of which are known to be dysregulated in the disease pathogenesis.

### 1.8. Versican and Aggrecan in PAH

Versican and aggrecan both belong to a PG family called lecticans. These chondroitin sulfate proteoglycans (CSPGs) also contain DS and KS, are found in the ECM, and are the largest and most highly glycosylated PGs. Aggrecan is the best studied member of this group due to its abundance in cartilage and brain and has the ability to withstand compressive forces as well as changes in water composition (desorption/resorption) [154]. Versican is predominantly found in the pericellular and interstitial ECM of connective tissues, blood vessels, brain, and leukocytes [154] and has been documented for its role in cell adhesion, migration, and proliferation [207,208]. Interestingly enough, both versican and aggrecan can form super molecules with HA in the ECM. Recently, levels of CSPGs were found to be high in PAH PASMCs. In particular, versican was shown to be increased in medial thickened regions, the neointima, and in the plexiform lesions as well as overall lung tissues of patients with IPAH [209]. Aggrecan was also found in the intima, media, and adventitia of both small and large IPAH vessels [210]. These data suggest that CSPGs may have a pathogenic role in PAH and may be involved in vascular remodeling and cell proliferation.

### 1.9. Other PGs in PAH

Syndecan is a PG that contains both HS and CS GAGs and is made up of four members (Syndecan 1–4). These PGs are membrane bound and are found in most nucleated cells [154]. Syndecan has been shown to be dysregulated in several cancers and facilitates cell adhesion, migration, and actin cytoskeletal organization as well as to interact with multiple growth factors and matrix components [211,212,213]. Syndecan can be cleaved, shedding an active ectodomain containing GAGs, which retain potent biological activity [214,215,216]. It was recently reported that while plasma levels of Syndecan-1 (along with other GAGs/PGs) increased in monocrotaline (MCT)-induced PAH rats, they decreased in the pulmonary arteries compared to wild-type controls [217], suggesting destruction of the pulmonary vascular glycocalyx. Other reports have shown that prolargin, which interacts with PGs (and does not contain GAGs), has some potential in differentiating PH subtypes based on the level of pulmonary vascular remodeling [218], indirectly suggesting that PGs are involved in the disease remodeling process. Overall, these data suggest that Syndecan, as well as other PG interacting proteins, may regulate vascular remodeling and contribute to overall PAH disease severity.

### 1.10. Galectins (Carbohydrate Lectins) in PAH

Galectins (Gal) are β-galactoside-binding lectins expressed in several cells types and tissues [219]. They are involved in immune responses such as inflammation, metabolism, wound healing, autophagy, signaling, and angiogenesis [220,221,222,223,224]. Currently, there are 15 different galectins that have been identified. They have been classified based on their biochemical structure into three groups: (1) Gals-1, -2, -5, -7, -10, -11, -13, -14, and -15 exists as monomers or dimers and display a single carbohydrate-recognition domain (CRD); (2) Gals-4, -6, -8, -9, and -12 form tandem-repeats and have two CRDs with a linker peptide; and (3) Gal-3 (chimera-type) CRD and an non-lectin domain with the ability to oligomerize [225]. 

Gal-1 and -3 have been studied in cancer, fibrosis, infection, and auto-immune disease and are known to interact with signaling molecules, transcriptional regulators, lysosomal proteins, cell surface receptors, and ECM proteins through glycans on the surface of these proteins [224,225,226,227]. They have also been shown to promote angiogenesis through interaction with a specific glycoform of vascular endothelial growth factor receptor (VEGFR) [228,229] and have been studied in cardiovascular disease [230,231]. 

In PAH, the majority of the investigations involve the functional consequences of increased Gal-3 in the disease [232,233,234,235]. This may be due to the uniqueness of Gal-3 and its single classification, limiting redundancy from other isoforms during the investigation. Nevertheless, Gal-1 has also been studied in PAH [236] though there is no literature exploring the other galectin isoforms in the disease. Interestingly, direct investigation of carbohydrate-galectin interactions has not been studied in PAH. It is worth postulating that these carbohydrate ligands may be affected by the same environmental cues (e.g., nutrient levels, ROS, etc.) that modulate metabolic pathways in PAH. In particular, the alterations in glycan machinery (glycosyltransferases and glycosidases), levels of sugar nucleotide substrates, and overall glycan composition could be similar to our findings with OGT, UDP-GlcNAc, and O-GlcNAc in IPAH [150]. Therefore, strategies to determine the changes in glycan as well as the glycosyltransferases/hydrolases involved are likely to be of interest. 

### 1.11. Sialylation in PAH 

Not much is known about the role of sialylation in PAH. Recently, Morrow et al. reported in a FASEB journal abstract that IgG sialylation/glycosylation was increased in PAH patients, affecting IgG binding to endothelial cells [237]. Free sialic acid, which is a signature found in cancer and cardiovascular disease, was also suggested to be increased in the serum of PAH patients compared to controls in another abstract by Morrow et al. [238]. Other studies showed that sialylation of the von Willebrand factor, a known marker for worse survival rates in patients with PAH [239], was reduced in PAH (or precapillary PH) [240]. These data suggest that altered sialic acid levels in PAH may have a role in the disease pathogenesis; specifically, terminal sialylation of glycoproteins can influence the binding and function galectins as well as other carbohydrate lectins.

## 2. Conclusions

Pulmonary hypertension has key features of progressive thickening and remodeling of the vasculature caused by endothelial dysfunction and aberrant pulmonary vascular cell proliferation. Tremendous effort has been put into investigating the pathobiology of PAH and key features that could reverse/slow the disease process. In particular, there is a need to explore more therapeutic options other than vasodilation in PAH. Our knowledge of metabolic dysfunction in PAH has advanced over the years; however, there are still important molecular details regulated by metabolic changes that we do not fully understand, and investigating glycans and their role in PAH may hold key details.

**Figure 1:**
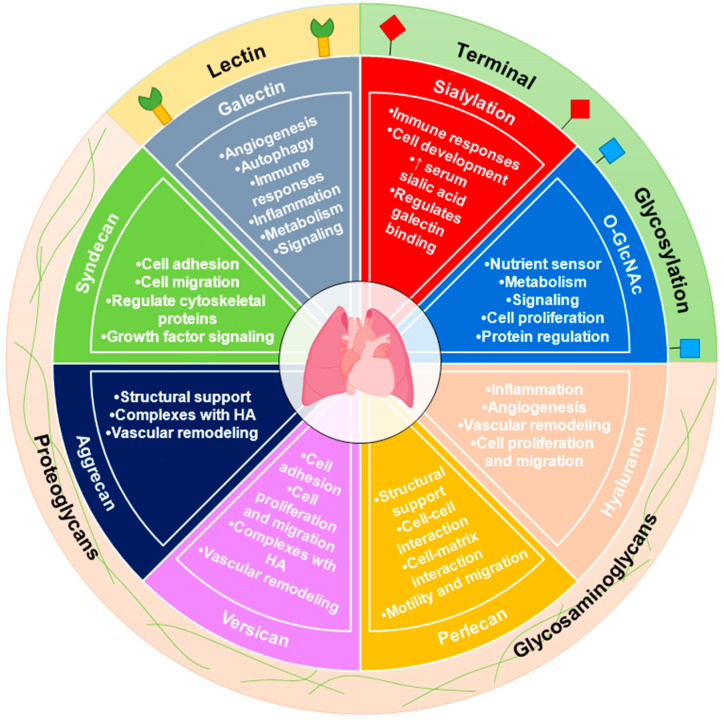
The glycobiology of PAH. Changes in glycosylation have been linked to dysregulated cellular metabolism, a hallmark of both PAH and cancer. Galectins (Lectins); O-GlcNAc and sialylation (Terminal Glycosylation); as well as hyaluronan, perlecan, versican, aggrecan, and syndecan (Proteoglycans and Glycosaminoglycans) have been investigated in PAH.

It is clear from past reports in cancer as well as recent reports in PAH that aberrant glycosylation can cause or perpetuate disease processes (Figure 1). Intracellular glycosylation such as the O-GlcNAc modification is highly dynamic, and its tight regulation by the cell is important in PAH. Targeting the O-GlcNAc transferase or the O-GlcNAc hydrolase may help to slow or reverse the metabolic changes. Indeed, the O-GlcNAc modification is important in the regulation of specific PAH cellular phenotypes and more studies are needed to determine specific proteins that may be under the control of the O-GlcNAc modification in the disease.

PGs/GAGs (HA) and galectins are increased in PAH. In addition, sialylation may be altered. However, there specific roles and how they contribute to vascular remodeling and regulation of vascular function as well as cell proliferation, cell signaling, and cell differentiation/morphology have not been defined. Are the changes observed in these glycans/glycoconjugates a consequence of the metabolic derangements observed in PAH? Or do these glycoconjugates, when altered, have a direct role in the PAH pathogenesis? Studying the specific role of glycans and/or glycoconjugates is critical to fully understanding the molecular mechanisms associated with dysregulated metabolism in PAH. These efforts cannot move forward without some technical challenges, which are contingent on the availability and accessibility of better tools for quantifying and precisely determining the roles of glycans and glycosylation events in cell biology. Targeted approaches to regulating glycosyltransferases/hydrolases, sugar nucleotides, and/or specific glycoconjugates/glycoforms are needed to advance our knowledge of pulmonary hypertension as well as other diseases defined by metabolic dysregulation.
